# Precise *ERBB2* copy number assessment in breast cancer by means of molecular inversion probe array analysis

**DOI:** 10.18632/oncotarget.12421

**Published:** 2016-10-03

**Authors:** Matthias Christgen, Jana L. van Luttikhuizen, Mieke Raap, Peter Braubach, Lars Schmidt, Danny Jonigk, Friedrich Feuerhake, Ulrich Lehmann, Brigitte Schlegelberger, Hans H. Kreipe, Doris Steinemann

**Affiliations:** ^1^ Institute of Pathology, Hannover Medical School, Hannover, Germany; ^2^ Institute of Human Genetics, Hannover Medical School, Hannover, Germany

**Keywords:** HER2/ERBB2, OncoScan, breast cancer, copy number profiling, next generation sequencing

## Abstract

HER2/*ERBB2* amplification/overexpression determines the eligibility of breast cancer patients to HER2-targeted therapy. This study evaluates the agreement between *ERBB2* copy number assessment by fluorescence *in situ* hybridization, a standard method recommended by the American Society of Clinical Oncology/College of American Pathologists (ASCO/CAP), and newly available DNA extraction-based methods. A series of n=29 formalin-fixed paraffin-embedded breast cancers were subjected to *ERBB2* copy number assessment by fluorescence *in situ* hybridization (FISH, Vysis, Abbott). Following macrodissection of invasive breast cancer tissue and DNA extraction, *ERBB2* copy number was also determined by molecular inversion probe array analysis (MIP, OncoScan, Affymetrix) and next generation sequencing combined with normalized amplicon coverage analysis (NGS/NAC, AmpliSeq, Ion Torrent). *ERBB2* copy number values obtained by MIP or NGS/NAC were tightly correlated with *ERBB2* copy number values obtained by conventional FISH (*r*_s_ = 0.940 and *r*_s_ = 0.894, *P* < 0.001). Using ASCO/CAP guideline-conform thresholds for categorization of breast cancers as HER2-negative, equivocal or positive, nearly perfect concordance was observed for HER2 classification by FISH and MIP (93% concordant classifications, κ = 0.87). Substantial concordance was observed for FISH and NGS/NAC (83% concordant classifications, κ = 0.62). In conclusion, MIP facilitates precise *ERBB2* copy number detection and should be considered as an ancillary method for clinical HER2 testing.

## INTRODUCTION

The HER2*/ERBB2* (erb-b2 receptor tyrosine kinase 2) proto-oncogene is activated by overexpression/amplification in 15% of breast cancers (BCs) [1]. The development of therapeutics targeting HER2 (trastuzumab, T-DM1, pertuzumab, lapatinib) has been a major breakthrough. According to current standard therapeutic regimes, *ERBB2* amplification is the only actionable genetic alteration in BC. Eligibility of BC patients to HER2-targeted therapy depends on detection of HER2/*ERBB2* overexpression/amplification in the tumor tissue by immunohistochemistry (IHC) and/or *in situ* hybridization (ISH). This methodology was introduced in now historical clinical trials, such as HERA and NSABP B-31 [2, 3]. Detailed guidelines for clinical HER2 testing are provided by the American Society of Clinical Oncology/College of American Pathologists (ASCO/CAP) [4]. From a technical point of view, ASCO/CAP guidelines admit a number of methodological variations for clinical HER2 testing, including dark field fluorescent ISH (FISH), bright field chromogenic ISH (CISH), ISH with just one probe for *ERBB2* (chromosome 17q12) or ISH with two probes for *ERBB2* and the centromeric region of chromosome 17 (CEP17). ASCO/CAP guidelines also define thresholds for a *ERBB2* positive status, which take into consideration either the *ERBB2*/CEP17 ratio and/or the *ERBB2* copy number (CN) *per se* [4].

HER2/*ERBB2* assessment by IHC and FISH is subject to at least some inter-observer and inter-laboratory variability [5]. Moreover, BCs with equivocal results by IHC and ISH are notoriously difficult to classify with conventional methods [6–12]. Consequently, there is a vivid debate about ancillary or third line test methods [13]. So far, ASCO/CAP guidelines preclude mRNA-based *ERBB2* expression assays, for which partly encouraging results, but also occasional misclassifications have been reported [14–19]. Meanwhile, innovative and rapidly developing DNA extraction-based methods have begun to play a substantial role in the classification of tumors [20]. Clinical HER2 testing might take advantage of these developments, which have made available new DNA-based methods for gene CN assessment. Here, we adopted two innovative DNA-based technologies, namely molecular inversion probe array analysis (MIP) [21, 22] and next generation sequencing with normalized amplicon coverage analysis (NGS/NAC) [23] for *ERBB2* CN assessment in BC.

## RESULTS

**Figure 1:**
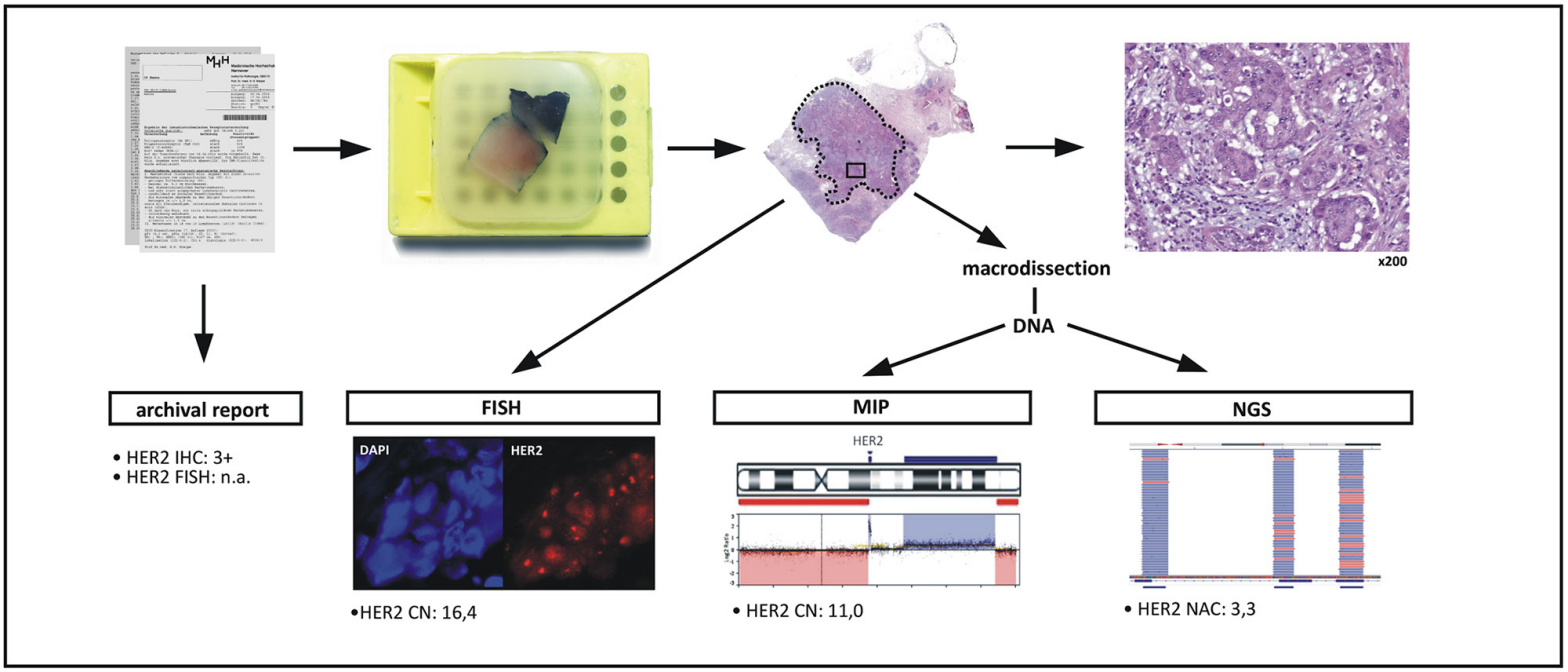
**Work flow:** An FFPE BC specimen was selected based on its HER2 status retrieved from archival reports. A representative FFPE tissue block was selected and was subjected to HER2/*ERBB2* copy number (CN) assessment by fluorescence *in situ* hybridization (FISH). Following macrodissection of invasive tumor tissue (dotted line) and DNA extraction, the HER2/*ERBB2* CN was also assessed by molecular inversion probe array analysis (MIP) and next generation sequencing with normalized amplicon coverage analysis (NGS).

A series of n=29 BCs was subjected to *ERBB2* CN assessment by FISH, MIP and NGS/NAC. FISH served as an ASCO/CAP guideline-conform standard method and was performed on whole-slide sections. No significant intratumoral heterogeneity for *ERBB2* was observed by FISH in any of the BCs included. MIP and NGS/NAC were performed with DNA from macro-dissected tumor tissue. An average *ERBB2* CN value was obtained by FISH and MIP. A *ERBB2* NAC value, reflecting the average *ERBB2* gene dosage, was obtained by NGS/NAC (Figure 1 and Table 1).

**Table 1:** BC characteristics in detail

			data from archival reports										*ERBB2* re-evaluation in the present study, same FFPE block					
case	ID	age	side	histology	pT	pN	grade	Ki67	ER	PR	HER2	*ERBB2* FISH	FISH [CN]		MIP [CN]		NGS [NAC]	
1	2	74	L	8500/3	1c	x	3	20	1	1	2	equivocal	4,1	equivocal	4,00	equivocal	0,55	negative
2	3	40	L	8500/3	2	x	3	25	1	0	3	n.a.	22,9	positive	11,67	positive	3,13	positive
3	4	35	L	8500/3	3	3a	3	65	1	1	3	n.a.	16,4	positive	11,00	positive	3,28	positive
4	6	77	L	8500/3	x	x	3	10	1	0	3	n.a.	18,5	positive	11,67	positive	4,22	positive
5	7	83	L	8500/3	2	1a	3	40	1	1	0	n.a.	2,0	negative	2,00	negative	0,48	negative
6	8	51	R	8500/3	1b	0	3	30	1	1	0	n.a.	1,9	negative	2,00	negative	0,50	negative
7	9	53	R	8500/3	2	0	3	30	1	1	0	n.a.	2,1	negative	2,00	negative	0,49	negative
8	10	69	L	8520/3	2	0	3	30	1	0	2	negative	2,0	negative	2,00	negative	0,37	negative
9	11	47	L	8500/3	1c	0	3	40	1	1	2	negative	3,7	negative	3,00	negative	0,97	negative
10	12	48	L	8500/3	3	3	3	30	1	1	0	n.a.	1,9	negative	1,67	negative	0,36	negative
11	13	38	L	8510/3	2	0	3	80	1	1	2	equivocal	2,9	negative	2,00	negative	0,59	negative
12	15	53	L	8500/3	1c	0	3	25	1	1	3	n.a.	7,6	positive	6,00	positive	0,88	negative
13	16	68	L	8500/3	1c	0	3	40	1	1	3	n.a.	11,2	positive	8,00	positive	1,12	negative
14	17	85	R	8500/3	1c	1	3	35	1	1	3	n.a.	19,9	positive	7,67	positive	2,44	positive
15	18	70	R	8500/3	4b	1	3	70	1	0	3	n.a.	11,5	positive	14,00	positive	2,29	positive
16	19	55	R	8500/3	2	1a	3	60	1	1	3	n.a.	24,6	positive	10,33	positive	3,42	positive
17	22	57	R	8500/3	2	0	3	25	1	1	2	negative	2,5	negative	3,00	negative	0,52	negative
18	23	65	L	8500/3	1c	x	2	50	1	1	2	equivocal	4,1	equivocal	5,00	equivocal	0,81	negative
19	24	51	R	8500/3	4b	0	3	35	1	1	2	equivocal	2,0	negative	2,00	negative	0,37	negative
20	25	64	R	8500/3	1b	0	2	10	1	1	2	negative	2,4	negative	2,33	negative	0,57	negative
21	26	73	L	8500/3	1b	0	3	20	1	1	0	n.a.	3,7	negative	2,70	negative	0,63	negative
22	27	40	R	8500/3	1c	0	3	10	1	1	2	negative	3,7	negative	4,00	equivocal	0,85	negative
23	28	56	R	8500/3	2	0	2	20	1	1	2	negative	2,1	negative	2,00	negative	0,33	negative
24	29	45	L	8500/3	1c	0	3	30	1	1	2	equivocal	2,8	negative	2,50	negative	0,52	negative
25	30	53	R	8520/3	3	0	2	15	1	1	0	n.a.	1,6	negative	1,00	negative	0,34	negative
26	31	65	R	8520/3	2	0	2	15	1	1	0	n.a.	2,1	negative	2,00	negative	0,36	negative
27	32	57	L	8520/3	3	0	2	5	1	1	2	equivocal	4,9	equivocal	7,00	positive	0,73	negative
28	33	64	R	8500/3	2	0	2	15	1	1	0	n.a.	2,2	negative	2,00	negative	0,33	negative
29	34	54	R	8500/3	2	0	3	30	1	1	0	n.a.	2,8	negative	2,00	negative	0,36	negative

**Figure 2:**
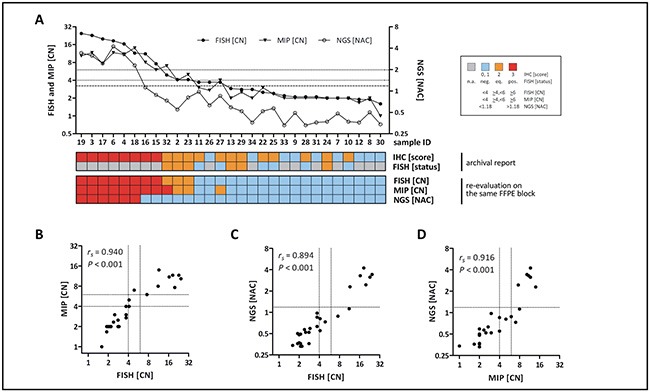
HER2/*ERBB2* CN detection by three methods (FISH, MIP, NGS/NAC) **A.** Diagrammatic representation of all cases ordered by FISH-based CN. Dotted lines indicate thresholds implemented for FISH and MIP. The dashed line indicates the threshold implemented fro NGS/NAC. **B.** Correlation of HER2/*ERBB2* CN values obtained by FISH and MIP **C.** Correlation of HER2/*ERBB2* CN values obtained by FISH and NGS/NAC **D.** Correlation of HER2/*ERBB2* CN values obtained by MIP and NGS/NAC. Dotted lines indicate thresholds used to classify HER2 as negative, positive or equivocal.

*ERBB2* CN or NAC values obtained by MIP or NGS/NAC were tightly correlated with *ERBB2* CN values obtained by FISH (Spearman correlation coefficients *r_s_* > 0.890, *P* < 0.001) (Figure 2). Counting of *ERBB2* FISH signals in the tumor cells and *ERBB2* CN values obtained by MIP showed a particularly tight correlation (*r_s_* = 0.940, *P* < 0.001) and an excellent agreement on a case-by-case basis (Figure 2A). FISH obtained slightly higher CN values in those cases with high level amplification (Figure 2A).

Next, the HER2 status of each BC was categorized as negative, positive or equivocal, using ASCO/CAP 2013 CN thresholds for FISH and MIP data [4]. For NGS/NAC values, a provisional cutoff was implemented as detailed in the material and methods section. Nearly perfect concordance was observed for HER2 classification by FISH *versus* MIP (93% concordant classifications, κ = 0.87) (Table 2). Substantial concordance was observed for HER2 classification by FISH *versus* NGS/NAC (83% concordant classifications, κ= 0.62) (Table 2).

**Table 2:** Concordance of *ERBB2* classification by CN

	FISH					MIP				
	neg	eq	pos	concordance	kappa	neg	eq	pos	concordance	kappa
MIP				27/29 (93%)	0.87					
neg	17	0	0							
eq	1	2	0							
pos	0	1	8							
NGS				24/29 (83%)	0.62				23/29 (79%)	0.56
neg	18	3	2			17	3	3		
eq	0	0	0			0	0	0		
pos	0	0	6			0	0	6		

## DISCUSSION

This is the first study, which determined agreement between MIP- or NGS/NAC-based *ERBB2* CN assessment and FISH-based *ERBB2* CN assessment in BC. Notably, MIP achieved nearly perfect agreement with FISH. Although this study was limited to comparatively few cases, our finding renders MIP a strong candidate for routine diagnostic application. A practical advantage of MIP is that ASCO/CAP CN thresholds are directly applicable to the data format generated by MIP.

NGS/NAC showed a slightly poorer performance. Two cases (IDs #15 and #16), were HER2/*ERBB2*-positive by IHC (3+), FISH, and MIP, but were classified as HER2-negative by NGS/NAC. Notably, these cases were closely above the CN threshold implemented for FISH and MIP and just below the CN threshold used for NGS/NAC. Of six cases showing an equivocal HER2 status by routine diagnostic assessment (IHC 2+/FISH-equivocal, namely IDs #32, #2, #23, #13, #29, and #24), three were confirmed as HER2 equivocal by repeated FISH in the context of this study. Two cases were confirmed as HER2 equivocal by MIP as well (IDs #2 and #23), one was assessed as HER2-positive by MIP (ID #32) showing a CN of 7 according to the TuScan algorithm, whereas positivity is defined as a CN ≥6. However, all these six cases were HER2-negative by NGS/NAC. Thus, NGS/NAC, as performed in this study, had a limited sensitivity for detection of borderline CN alterations and low level *ERBB2* amplification. The performance of HER2 classification by NGS/NAC may be improved by fine-tuning of the NAC threshold defining *ERBB2* positivity. This, however, requires additional studies in larger cohorts of HER2-positive BCs and HER2-negative controls. Clinical patient selection for HER2-targeted therapy should not be based on NGS/NAC analysis to this point.

An advantage of MIP and NGS/NAC is the possibility to determine *ERBB2* CN and hot spot mutations in cancer related genes, such as *PIK3CA* or *TP53*, simultaneously (data no shown). Moreover, MIP assays generate genome-wide CN information. This may become clinically relevant, because distinct somatic mutations and CN variations may predict resistance to HER2-targeting therapy [25, 26]. The ability to gain genome wide CN variation information makes MIP a very strong method for *ERBB2* CN classification and probably for patient stratification too. A potential disadvantage of MIP and NGS/NAC is that intratumoral heterogeneity for *ERBB2* amplification could result in false-negative findings. According to ASCO/CAP guidelines, *ERBB2* amplification in just 20 adjacent BC cells suffices to establish a HER2-positive status, even if all other parts of the tumor are negative for HER2 protein expression and *ERBB2* amplification [4]. No such intratumoral heterogeneity was observed in the BC cases included in this study. However, DNA extraction-based methods certainly fail to classify such cases as *ERBB2*-positive. Intratumoral heterogeneity is often stressed in debates concerning *in situ* and extraction methods. Importantly, HER2-positive subclones do not dictate the course of the disease [15, 27].

BCs with an equivocal test result by IHC and ISH account for 5% to 15% of all BC cases [6–12]. ASCO/CAP guidelines warrant repeated testing by IHC or ISH in these instances and preclude alternative test types, such as mRNA-based *ERBB2* expression assays [4]. Variable results and occasional misclassifications have been reported for Oncotype DX, a 21-gene-qRT-PCR expression test including *ERBB2* [14, 16]. *ERBB2* mRNA analysis has remained at least partly controversial, despite individual studies have documented substantial to almost perfect overall agreement between HER2 assessment by mRNA expression assays and conventional methods (κ= 0.73 – 0.84) [15, 17–19]. Detection of *ERBB2* gene amplification is also possible by RNA *in situ* quantification using RNAscope and was shown to be superior to qPCR in cases with equivocal FISH results or intratumoral heterogeneity [28, 29]. Given the problematic acceptance of HER2 classification by mRNA expression analysis, MIP would represent an ideal ancillary test method, especially for cases with a HER2-equivocal status. First, MIP and ISH assess the same substrate (DNA). Second, *ERBB2* CN assessment by ISH is limited to what is feasible to count, but MIP pools ten thousands of tumor cells and has the power to objectify or rectify prior IHC/ISH test result. In summary, this is the first study showing that MIP facilitates precise *ERBB2* CN assessment in BC. MIP should be considered as an ancillary test type for clinical HER2 diagnostics.

## MATERIALS AND METHODS

### BC specimens and DNA extraction

Formalin-fixed paraffin-embedded (FFPE) BC resection specimens were selected based on the HER2 status established by ASCO 2013 guideline-conform routine diagnostic assessment (RDA). To obtain a homogenous sample collection, cases were restricted to estrogen receptor (ER)-positive BCs. To support subsequent correlation analyses for *ERBB2* CN, the series was evenly composed of HER2-positive, HER2-negative and HER2-equivocal cases (Table 3). All specimens were retrieved from the archive of the Institute of Pathology of the Hannover Medical School according to the guidelines of the local ethics committee and were made anonymous for scientific purposes. Subsequently, *ERBB2* CN was assessed by FISH, MIP and NGS on the very same FFPE tissue block for all three assays. The HER2 IHC status was adopted from archival reports (Figure 1). For MIP and NGS, total DNA was extracted from macrodissected invasive tumor tissue (n=16 sections per case, 8 μm each). To guide macrodissection, an experienced pathologist (m.c.) encircled the entire invasive tumor with a pen on an extra HE-stained section taken from the middle of the section series cut for DNA extraction. Areas with carcinoma *in situ* were spared. DNA extraction was carried out with the DNeasy blood and tissue kit (Qiagen, Hilden, Germany) according to the manufacturers recommendations, with slight modifications. In brief, the xylol/ethanol proportion was slightly modified to1200 μl (instead of1000 μl) and, following resuspension of the pellet in ALT buffer, an additional incubation for 15 min at 98°C was included. Moreover, following proteinase K digest, an additional incubation in RNase A (250 μg/ml, AppliChem, Darmstadt, Germany) for 30 min at 37°C was included. DNA amount was quantified with a Qubit fluorometer (Thermo Fisher Scientific, Waltham, MA., U.S.A). A total of 29 out of 34 initially selected cases yielded sufficient DNA amount and quality for all assays (Table 3). An additional set of n=12 FFPE normal tonsils were included as controls for NGS/NAC analysis (see below).

**Table 3:** BC characteristics

	number	percent
all cases	29	100
age		
<60	17	57
>60	12	43
pT stage		
pT1	11	38
pT2	11	38
pT3/4	6	21
pTx	1	3
pN stage		
pN0	19	65
pN1+	6	21
pNx	4	14
grade		
G1/G2	7	24
G3	22	76
ER		
negative	0	0
positive	29	100
PR		
negative	4	14
positive	25	86
HER2*		
negative (IHC 0, 1+, FISH n.a.)	9	31
negative (IHC 2+, FISH -)	6	21
equivocal (IHC 2+, FISH equivoval)	6	21
positive (IHC 3+, FISH n.a.)	8	27

* HER2 status from archival reports

### Fluorescence *in situ* hybridization (FISH)

FISH was performed on an extra whole slide FFPE sections (5 μm) using the Vysis PathVysion HER2 probe (Abbott, Wiesbaden, Germany). Two observer independently quantified *ERBB2* signals in 50 tumor cells each. Clusters of *ERBB2* signals were scored with an estimated *ERBB2* CN per cluster. The average *ERBB2* CN per cell was calculated as the sum of the counts of the two observers divided by 100. CN thresholds used for the definition of a negative, equivocal or positive HER2 status were <4, ≥4 to <6 and ≥6, as recommended by ASCO/CAP 2013 guidelines [4].

### Molecular inversion probe (MIP) DNA array analysis

MIP array analysis was performed with 80 ng DNA and OncoScan® arrays following protocols provided by manufacturer (Affymetrix, Santa Clara, (CA), USA). Briefly, samples were split to separate (A/T) and (G/C) channels. After circularization, MIPs were linearized, cleaved and were then amplified by PCR. Amplicons were cleaved into two fragments (44 bp) with *HaeIII*. DNA fragments were subsequently hybridized to OncoScan® arrays at 58°C for 18 h. Next, arrays were stained and washed using the GeneChip® Fluidics station 450, and were scanned using the GeneChip® scanner 3000 7G (Affymetrix). Array fluorescence intensity (CEL) files were generated with Affymetrix® GeneChip® Command Console® (AGCC, Affymetrix). CEL files were processed with OncoScan Console software version 1.3.0.39 to produce OSCHP files and QC metrics. Samples passing QC criteria (MAPD ≤ 0.3, ndSNPQC ≥ 26) were further analyzed using OncoScan® assay SM calls and Chromosome Analyses Suite (ChAS) version 3.1.0.15 (r9069) for CN variation. The *ERBB2* CN was determined with the TuScan algorithm. Based on B-allele frequencies (BAFs) and log2-ratios, the TuScan algorithm provides CN values adjusted for the estimated ploidy and percentage of aberrant cells included in the sample. However, if the sample is highly heterogeneous, TuScan provides average CN values. CN thresholds used for the definition of a negative, equivocal or positive HER2 status were adopted from ASCO/CAP 2013 ISH guidelines. A *ERBB2* CN of <4 was considered negative, a CN of ≥4 to <6 was considered equivocal and a CN of ≥6 was considered positive [4]. The complete data series is deposited at GEO under the accession number GSE83916 (http://www.ncbi.nlm.nih.gov/geo/query/acc.cgi?acc=GSE83916).

### Next generation sequencing (NGS) and normalized amplicon coverage (NAC) analysis

Library preparation was performed with Ion AmpliSeq library kit 2.0. Quantification of prepared libraries was conducted by qPCR using the Ion Library Quantification Kit. For template preparation using the Ion OneTouch 2 instrument, 12 BC samples were pooled (100 pM each). Sequencing was performed with Ion PGM Hi-Q Kit v2 and using 318 v2 Chips. Analyses of sequencing raw data were performed with Torrent server software (version 4.2.1). The mean sequencing depths for the n=29 BCs was 3869 reads. The mean sequencing depths for the n=12 tonsil specimens, which served to define a NAC cutoff for *ERBB2* positivity, was 2484 reads. For the calculation of the normalized amplicon coverage, the read count for a given amplicon was divided by the mean read count for this sample. The mean value for the three amplicons covering *ERBB2* was calculated. The same value was calculated for the 8 amplicons covering *TP53* and the ratio of these two values was computed. The mean value plus two times the standard deviation obtained from the n=12 tonsil samples (=1.18) was implemented as a threshold defining an *ERBB2* CN gain.

### Statistics

Correlation analyses were performed with GraphPad Prims software (version 5, Graph Pad Inc. San Diego, U.S.A). Concordance of *ERBB2* classification was assessed with JMP Pro10 software (SAS, Marlow, UK) and Cohens unweighted κ [24].
